# Wrapping the pancreas with a polyglycolic acid sheet before stapling reduces the risk of fluid collection on the pancreatic stump after distal pancreatectomy

**DOI:** 10.1007/s00464-021-08387-0

**Published:** 2021-02-23

**Authors:** Ji Su Kim, Seoung Yoon Rho, Dong Min Shin, Munseok Choi, Chang Moo Kang, Woo Jung Lee, Ho Kyoung Hwang

**Affiliations:** 1 Pancreatobiliary Cancer Center, Yonsei Cancer Center, Severance Hospital, Seoul, Korea; 2 Department of Hepatobiliary and Pancreatic Surgery, Yongin Severance Hospital, Yongin, Korea; 3 Department of Hepatobiliary and Pancreatic Surgery, Yonsei University College of Medicine, 50-1 Yonsei-ro, Seodaemun-gu, Seoul, 03722 Korea

**Keywords:** Distal pancreatectomy, Polyglycolic acid, Pancreatic fistula, Fluid collection, Stapling

## Abstract

**Background:**

Postoperative pancreatic fistula (POPF) and postoperative fluid collection (POFC) are common complications after distal pancreatectomy (DP). The previous method of reducing the risk of POPF was the application of a polyglycolic acid (PGA) sheet to the pancreatic stump after cutting the pancreas with a stapler (After-stapling); the new method involves wrapping the pancreatic resection line with a PGA sheet before stapling (Before-stapling). The study aimed to compare the incidence of POPF and POFC between two methods.

**Methods:**

Data of patients who underwent open or laparoscopic DPs by a single surgeon from October 2010 to February 2020 in a tertiary referral hospital were retrospectively analyzed. POPF was defined according to the updated International Study Group of Pancreatic Fistula criteria. POFC was measured by postoperative computed tomography (CT).

**Results:**

Altogether, 182 patients were enrolled (After-stapling group, *n* = 138; Before-stapling group, *n* = 44). Clinicopathologic and intraoperative findings between the two groups were similar. Clinically relevant POPF rates were similar between both groups (4.3% vs. 4.5%, *p* = 0.989). POFC was significantly lesser in the Before-stapling group on postoperative day 7 (*p* < 0.001).

**Conclusions:**

Wrapping the pancreas with PGA sheet before stapling was a simple and effective way to reduce POFC.

**Supplementary Information:**

The online version contains supplementary material available at 10.1007/s00464-021-08387-0.

Although the morbidity and mortality rates after pancreatic surgery have decreased significantly in recent years due to the development of surgical techniques and improved perioperative management, postoperative pancreatic fistula (POPF) is still the most common and serious complication that cannot be easily resolved [1]. POPF after distal pancreatectomy (DP) is not as severe as that after pancreatoduodenectomy (PD); however, it is associated with other complications, including intra-abdominal abscess, intra-abdominal hemorrhage, delayed gastric emptying, and sepsis [2, 3]. Postoperative fluid collection (POFC) is also frequently observed after DP.

Several studies have suggested ways to reduce the incidence of POPF after DP, including hand-sewn closure, main-duct isolated ligation, transection, and closure using a stapling device, pancreatic transection using energy devices, fibrin sealants, and the use of patches or sheets [1, 4–9]. Despite much effort, the clinically relevant POPF (CR-POPF) rate after DP remains between 4 and 40%. Recently, as minimally invasive surgery has increased when performing DP in pancreatic cancer as well as benign to borderline tumors of the pancreas, it seems that cutting the pancreas using a stapler is one of the most commonly used methods [10]. In addition, Jang et al. introduced a simple method in their randomized clinical trial to reduce the risk of POPF by applying a polyglycolic acid sheet (PGA, Neoveil®, Gunze, Japan) to the pancreatic stump after cutting the pancreas using a stapler [11]. Neoveil® is a bio-absorbable (within 15 weeks) reinforcement material made from PGA.

In the past, we also attached a PGA sheet on the pancreatic stump after cutting the pancreas with a stapler (After-stapling method), but recently, we have used the technique of wrapping the pancreas with a PGA sheet first and then stapling on it (Before-stapling method) (Fig. 1). Since there are no studies to evaluate the efficacy of reducing POPF and POFC according to the time of applying PGA sheet, this study aimed to compare the incidence of POPF and POFC between the After-stapling and Before-stapling methods.

**Fig. 1:**
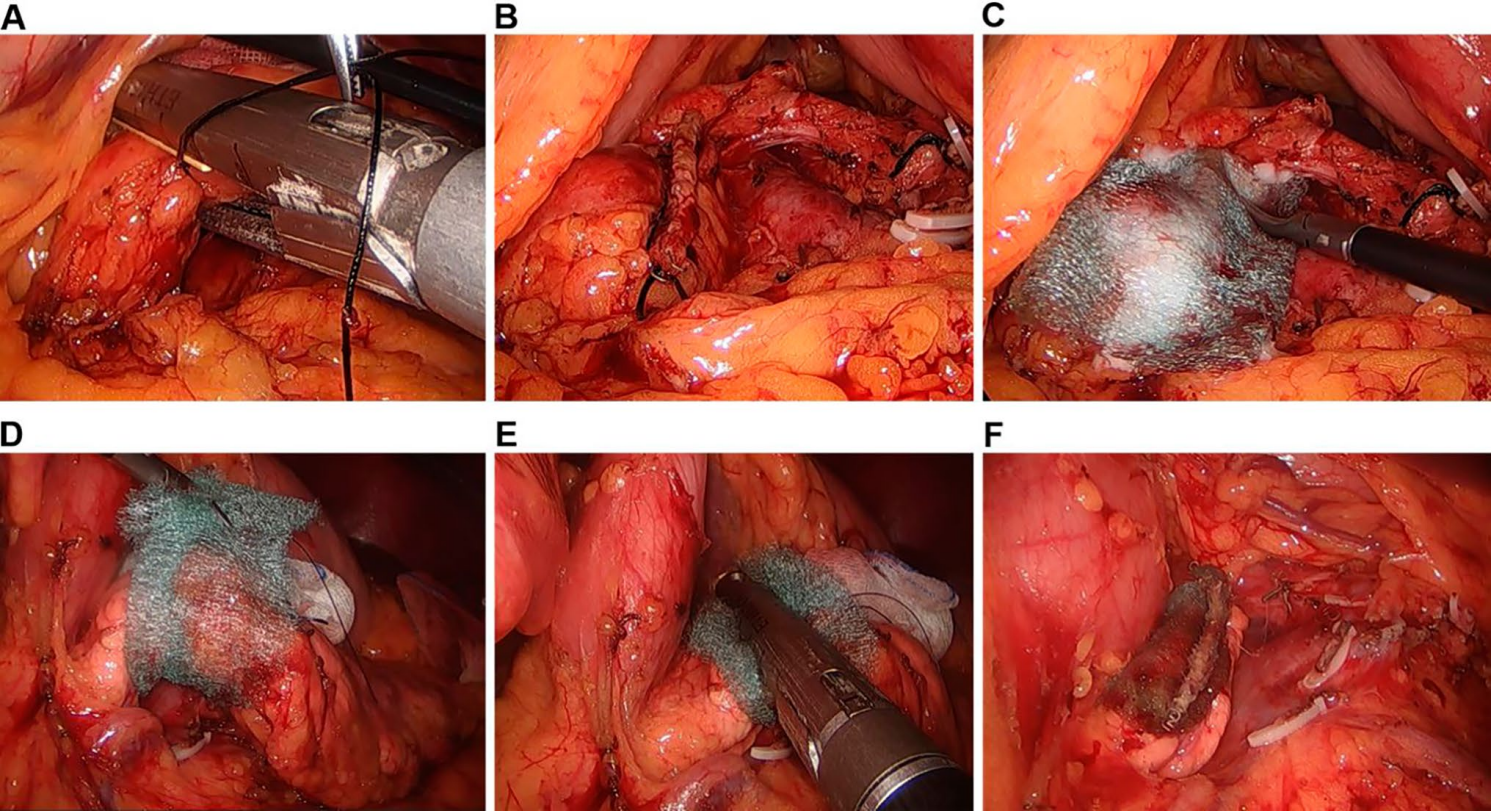
Two different methods of applying a polyglycolic acid sheet (PGA, Neoveil®) to the pancreatic stump. In the After-stapling group (**A**–**C**), the PGA sheet was attached on the pancreatic stump after cutting the pancreas with a stapler. However, in the Before-stapling group (**D**–**F**), the pancreas was first wrapped with a PGA sheet before stapling, and then the pancreas was cut using a stapler

## Methods

### Study population and surgical procedure

This retrospective study comprised patients who underwent open or laparoscopic DP by a single surgeon from October 2010 to February 2020 in a tertiary referral hospital. Case report forms were used to record the patients’ demographic characteristics (age, sex, body mass index [BMI], pathologic diagnosis, operative method, spleen preservation, operative time, intraoperative blood loss, postoperative hospital stay duration, and postoperative complications). All consecutive cases of DPs were included in a prospectively recorded database. The pancreas was only transected using an Endo-GIA™ (endovascular gastrointestinal anastomosis stapler, Covidien, North Haven, CT, USA) stapler in both open and laparoscopic surgeries. The thickness and length of the cartridge of the Endo-GIA were selected by the surgeon, with consideration of the thickness, length, and texture of the pancreas. The fibrin sealants were applied to the resection margin of the pancreas in all patients. The patients were classified into the following two groups according to when the PGA sheet was applied: the After-stapling group, in which the PGA sheet was attached on the pancreatic stump after cutting the pancreas with a stapler, and the Before-stapling group, in which the pancreas was first wrapped with a PGA sheet, and then the pancreas was cut using a stapler (Fig. 1). A closed suction silicon drain was placed near the pancreatic stump. The amount of fluid in the closed suction drain was checked daily. The amylase and lipase levels in the serum and intraperitoneal fluid obtained from a closed drain were regularly checked. This study was approved by the Institutional Review Board of Yonsei University College of Medicine (approval number: 4-2020-0393).

### Definition of variables and postoperative follow-up

**Fig. 2:**
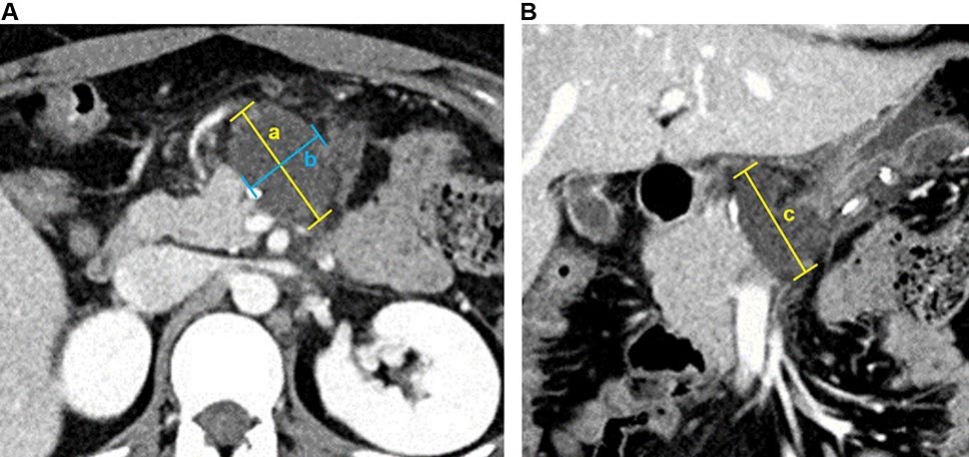
Measurement of the postoperative fluid collection on a computed tomography (CT) image. Among the multiple cut images of the postoperative CT scan, the image with the largest fluid collection was selected. The length (*a*) and width (*b*) on the axial view (**A**) and the height (*c*) on the coronal view (**B**) were measured. The volume of the postoperative fluid collection was calculated by multiplying the length, width, and height (volume of fluid collection = *a* × *b* × *c*, mm^3^)

POPF was defined according the updated International Study Group of Pancreatic Fistula (ISGPF) criteria established in 2016 that was retrospectively applied to all patients who underwent DP before 2016 [12]. A routine abdominal computed tomography (CT) was performed between postoperative days (PODs) 5 and 7 during hospitalization (short-term imaging follow-up). The first visit to the out-patient clinic after discharge was made between 1 and 2 weeks after discharge, at which time a basic medical interview was held. Moreover, the routine examinations, including blood testing and abdominal CT, were performed on POD 90. The next regular follow-up evaluations in the out-patient clinic were performed every 3–6 months to monitor for loco-regional or systemic recurrence in patients with a borderline tumor or ductal adenocarcinoma of the pancreas. For the patients with chronic pancreatitis, routine laboratory tests and abdominal CT were performed to check the remnant pancreas for recurrence of pancreatic cancer. To compare the difference in POFC between the After-stapling and Before-stapling groups, POFC was measured by CT performed on PODs 5–7 and 90. The hepatic venous phase with 3-mm thickness contrast-enhanced axial and coronal images was reviewed using a picture archiving and communication system (PACS) workstation (Marosis M-view 5.4; Marotech, Seoul, South Korea) at the pancreatic resection margin. Among the multiple cut images of the postoperative CT, the image showing maximum fluid collection was selected. The length and width on the axial image and the height on the coronal image were measured. The volume of POFC was calculated by multiplying the length, width, and height (Fig. 2).

### Statistical analysis

Continuous variables are expressed as the mean ± standard deviation (SD). Differences in continuous variables between the two groups were tested using the Student’s *t* test. Categorical variables are expressed as number (percentage). The associations among different categorical variables were determined using the Chi-squared or Fisher’s exact test. Statistical significance was defined as *p* < 0.05. Statistical analyses were performed using SPSS 24.0 for Windows (SPSS Inc., Chicago, IL, USA).

## Results

### No differences in clinicopathologic findings and intraoperative outcomes

Of the 193 patients who underwent DP during the study period, 11 patients were excluded due to combined gastrointestinal surgery (stomach cancer, 6; colon cancer, 5); finally, 182 cases were enrolled in this study. Table 1 presents the clinicopathologic characteristics and intraoperative findings of the enrolled patients. There were 84 men and 98 women with a mean age of 58.3 ± 14.6 (range 17–86) years. The After- and Before-stapling groups comprised 138 and 44 patients, respectively.

**Table 1:** Clinicopathologic characteristics and intraoperative findings

	All patients (*n* = 182)	After-stapling group (*n* = 138)	Before-stapling group (*n* = 44)	*p*-value
Age, years	58.3 ± 14.6	57.7 ± 14.3	60.2 ± 15.5	0.329
Sex, *n* (%)				0.557
Male/female	84 (46.2)/98 (53.8)	62 (44.9)/76 (55.1)	22 (50.0)/22 (50.0)	
BMI, kg/m^2^	22.5 ± 3.5	22.6 ± 3.5	22.2 ± 3.5	0.545
Diagnosis, *n* (%)				0.364
PDAC	68 (37.4)	48 (34.8)	20 (45.5)	
IPMN	25 (13.7)	16 (11.6)	9 (20.5)	
SPT	23 (12.6)	17 (12.3)	6 (13.6)	
NET	18 (9.9)	14 (10.1)	4 (9.1)	
SCN	8 (4.4)	7 (5.1)	1 (2.3)	
MCN	10 (5.5)	10 (7.2)	0	
RCC metastasis	6 (3.3)	5 (3.6)	1 (2.3)	
Chronic pancreatitis	13 (7.1)	12 (8.7)	1 (2.3)	
Others	11 (6.0)	9 (6.5)	2 (4.5)	
Method of surgery, *n* (%)				0.426
Open surgery	76 (41.8)	59 (42.8)	17 (38.6)	
Laparoscopic surgery	86 (47.3)	62 (44.9)	24 (54.5)	
Laparoscopic attempted	20 (11.0)	17 (12.3)	3 (6.8)	
Spleen preservation, *n* (%)	45 (24.7)	38 (27.5)	7 (15.9)	0.120
Location of pancreatic resection, *n* (%)				0.423
Neck	98 (53.8)	72 (52.2)	26 (59.1)	
Body to tail	84 (46.2)	66 (47.8)	18 (40.9)	
Operative time, min	284.0 ± 86.6	286.7 ± 80.0	275.5 ± 105.2	0.456
Intraoperative blood loss, ml	284.5 ± 369.0	284.8 ± 387.5	283.4 ± 307.9	0.982

*PDAC* pancreatic ductal adenocarcinoma, *IPMN* intraductal papillary mucinous neoplasm, *SPT* solid pseudo-papillary tumor, *NET* neuroendocrine tumor, *SCN* serous cystic neoplasm, *MCN* mucinous cystic neoplasm, *RCC* renal cell cancer, Others including pancreatic tuberculosis, fibromatosis, intraluminal tubulopapillary tumor, accessory spleen, colon cancer metastasis, ectopic splenic cyst, recurred stomach cancer, liposarcoma, lymphoma, *POPF* postoperative pancreatic fistula

Values are *n* (%) or mean ± standard deviation

There were no differences in age, sex, and BMI between the two groups. The most common pathologic diagnosis was pancreatic ductal adenocarcinoma (37.4%), followed by intraductal papillary mucinous neoplasm (13.7%), solid pseudo-papillary tumor (12.6%), and neuroendocrine tumor (9.9%). There was no difference in the pathologic diagnosis between the two groups (*p* = 0.364). The surgical methods, classified as open, laparoscopic, and laparoscopic attempted surgeries, were not different between the two groups (*p* = 0.426). There were no differences in the frequency of spleen preservation (*p* = 0.120), location of pancreatic resection (*p* = 0.423), operative time (*p* = 0.456), and intraoperative blood loss (*p* = 0.982) between the two groups (Table 1).

### No differences in POPF rates and postoperative outcomes

Biochemical leakage occurred in 101 patients (55.5%). Eight patients (4.4%) had grade B POPF, but none had grade C POPF. According to the POPF grade, there was no difference in the POPF rates between the After-stapling group and Before-stapling group (biochemical leakage, 55.8% vs. 54.5%; grade B, 4.3% vs. 4.5%; *p* = 0.989). There were no differences in the volumes of fluid drained, according to the postoperative day (*p* = 0.430, *p* = 0.895, *p* = 0.428, *p* = 0.405), drain removal day (*p* = 0.749), and postoperative hospital stay (*p* = 0.614) between the two groups (Table 2).

**Table 2:** Postoperative findings between the After- and Before-stapling groups

	All patients (*n* = 182)	After-stapling group (*n* = 138)	Before-stapling group (*n* = 44)	*p*-value
Postoperative pancreatic fistula, *n* (%)				0.989
No	73 (40.1)	55 (39.9)	18 (40.9)	0.901
Biochemical leakage	101 (55.5)	77 (55.8)	24 (54.6)	0.884
Grade B	8 (4.4)	6 (4.3)	2 (4.5)	1.000
Grade C	0	0	0	
Drainage amount, ml				
POD 1	163.9 ± 168.5	158.4 ± 163.9	181.7 ± 183.7	0.430
POD 2	157.1 ± 155.1	156.2 ± 157.5	159.8 ± 148.8	0.895
POD 3	149.8 ± 181.9	143.8 ± 177.7	169.1 ± 195.6	0.428
POD#4	161.4 ± 207.9	154.1 ± 207.8	184.8 ± 209.0	0.405
Drain removal day, days	9.2 ± 8.5	9.3 ± 9.3	8.8 ± 4.9	0.749
Re-intervention, *n*				0.574
No	178 (97.8%)	134 (97.1%)	44 (100%)	
Yes	4 (2.2%)	4 (2.9%)	0	
Postoperative hospital stay, days	15.3 ± 14.3	15.6 ± 15.6	14.3 ± 9.1	0.614
Postoperative fluid collection, *n* (%)				
POD 7	169 (92.9)	130 (94.2)	39 (88.6)	0.309
POD 90	83 (45.6)	65 (47.1)	18 (40.9)	0.473
Amount of fluid collection on CT image, mm^3^				
POD 7	30,320.6 ± 60,104.2	37,423.2 ± 67,357.7	8044.1 ± 9011.7	< 0.001
POD 90	21,148.8 ± 66,552.3	22,364.6 ± 71,624.2	17,335.5 ± 47,746.3	0.664
Readmission, *n*				0.782
No	163 (89.6)	124 (89.9)	39 (88.6)	
Yes	19 (10.4)	14 (10.1)	5 (11.4)	

*POD* postoperative day, *CT* computed tomography

Values are *n* (%), mean ± standard deviation or median (interquartile range)

### Reduced POFC in the before-stapling group

POFC was observed in most patients (*n* = 169, 92.9%) on CT performed on POD 7. A new percutaneous drainage catheter was inserted in 4 patients of the After-stapling group because of ineffective drainage and increase in fluid collection volume during hospitalization. Even though none of the patients in the Before-stapling group received therapeutic interventions for POFC, there was no statistical difference in the rate of re-intervention between the two groups (2.9% vs. 0%, *p* = 0.574) (Table 2). Although the incidence of POFC on POD 7 did not differ between the two groups, the volume of POFC was significantly lower in the Before-stapling group than in the After-stapling group (37,423.2 ± 67,357.7 vs. 8,044.1 ± 9,011.7 mm^3^, *p* < 0.001). Even though POFC remained until POD 90 in approximately half of the patients (83, 45.6%), spontaneous regression was observed in all patients during the long-term follow-up period. There was no difference in the volume of POFC on POD 90 between the two groups. (*p* = 0.664) (Table 2). Twelve patients (10.1%) in the After-stapling group and 5 patients (11.4%) in Before-stapling group were readmitted to receive conservative management for their symptoms (abdominal pain, indigestion, fever, and wound problems). No patient was readmitted for re-intervention to treat POPF or POFC, and there was no difference in the readmission rate between the two groups (*p* = 0.782) (Table 2).

## Discussion

The current study proposed a method to minimize the incidence of POPF or POFC after DP. This study showed that POFC can be reduced more effectively by making simple changes to the method of attaching a PGA sheet to the pancreatic stump. Although the CR-POPF rate was not significantly different between the After- and Before-stapling groups, the incidence of POFC was further reduced when the pancreas was first wrapped with a PGA sheet before stapling, and then stapling on it to cut the pancreas. In terms of POPF, although no difference was observed in the CR-POPF rate between the technique of applying the PGA sheet before and after stapling (4.3% vs. 4.5%, *p* = 0.989), the CR-POPF rate in both groups was sufficiently low, which was comparable to the rates reported by other studies that applied the PGA sheet to the pancreatic stump.

Despite various efforts to reduce the CR-POPF rate after DP, the CR-POPF rate remains between 4 and 40%, and it is still unclear which method is the most effective for reducing the POPF rate [1, 4–9]. Using the PGA sheet is one of the effective methods to reduce the incidence of POPF, with the CR-POPF rate being approximately 4% in other studies applying the PGA sheet to the pancreatic stump after DP [5, 9]. However, Jang et al. reported, in their recent multicenter randomized clinical trial, that the CR-POPF rate was 11.4% in PGA sheet wrapping group [11]. In our study, only grade B POPF occurred in 8 patients (4.4%) out of the 182 patients studied, and there was no patient with grade C POPF; our results either did not differ or were better than those of other previous studies [1, 4–9]. Based on these results, regardless of whether the PGA sheet is attached to the pancreatic stump after stapling (After-stapling group) or the pancreas is transected with a stapler after wrapping the pancreas with the PGA sheet (Before-stapling group), it can be concluded that using the PGA sheet is effective in reducing the incidence of POPF.

Given that the tearing of the pancreatic parenchyma is related to POPF on the pancreatic stump, a few methods, such as reducing the closing speed of the stapler [13–16] or using a stapling device with a pre-attached PGA sheet [17], were introduced to reduce the risk of injury to the pancreatic parenchyma. Other studies have demonstrated that prolonged pre-firing compression for approximately 3–5 min with a stapler can help to minimize the risk of POPF by decreasing the pancreatic thickness sufficiently to effectively stapling the pancreas [14–16]. We routinely performed pre-firing compression for 3–5 min before stapling in all patients and also performed the incision slowly for 1–2 min during firing.

In our literature review, we found a few studies that utilized the same method as ours. Ochiai et al. [9] first described the PGA wrapping method with GIA. Their procedure was the same as our method; during DP, the PGA sheet was wrapped around the predictive staple line of the pancreas to reduce compression tissue damage by a stapler device before stapling. In some cases, they used the Echelon 60 with the PGA sheet stocking type, which was released in only some countries and was not available in Korea. According to their results, severe POPF occurred in 10 out of 37 (27%) patients in the group that did not use PGA, whereas, in the group that used PGA, the incidence of severe POPF was only 1 out of 26 (4%) patients, showing a statistical difference (*p* = 0.017). Yamashita et al. also introduced the linear stapling device with a pre-attached PGA sheet (Endo-GIA™ Reinforced Reload with Tri-Staple™; black reload; 60-mm long; Covidien Japan Inc. Tokyo, Japan). They reported that the grade B or C POPF in the PGA sheet (+) group occurred in only one patient (5%) among the 22 patients, with the rate being significantly lower compared to that of the PGA sheet (−) group (5% vs. 30%, *p* = 0.0216) [17]. Considering some possible mechanisms that PGA sheet can reduce the incidence of POPF or POFC, first, when the pancreas is wrapped with a PGA sheet and then stapling is performed, the main pancreatic duct and branch duct in the pancreatic parenchyma at the pancreatic stump could be more completely sealed by closing the narrow gap between the staple and the pancreatic parenchyma. The second possible mechanism is that wrapping the pancreas with a PGA sheet before stapling can reduce the crushing injury to the pancreatic parenchyma when closing the jaw of the stapler. However, it is still unclear why the amount of POFC was lower in the Before-stapling group, even though the rates of biochemical leakage and CR-POPF were similar in both groups. One possible mechanism is that the PGA sheet inserted into the pancreas parenchyma with the stapler cartridge in the Before-stapling group could help heal the torn part of the pancreatic stump. POPF was defined as a peritoneal amylase value > 3 times the normal upper limit on POD 3; however, abdominal CT was performed between PODs 5 and 7. During PODs 2 or 4, the healing process might be more effective due to the PGA sheet embedded in the parenchyma of the pancreas. However, further studies, including animal experiments, are necessary to clarify the mechanism. To prevent crushing injuries, it is also important to select the proper Endo-GIA cartridge according to the pancreatic thickness and texture. If the pancreatic tissue is thick, a higher cartridge should be selected [18]. Apart from selecting an appropriate cartridge according to the thickness of the pancreas, the texture of the pancreatic tissue also needs to be evaluated; if the texture of the pancreatic tissue is firm, it may result in a crushing injury at the resected line of the pancreas. When the pancreas is wrapped with a PGA sheet, such crushing injury can be reduced.

In addition to the method of attaching the PGA sheet to the cut surface of the pancreas, other methods for reinforcement of the resection line of the pancreas have been also introduced. Hamilton et al. reported a study comparing the CR-POPF rate between patients who underwent stapled transection of the pancreas alone and patients who underwent stapled transection with reinforcement using commercially available mesh devices (Seamguard, W. L. Gore, Flagstaff, AZ, or Peristrips Dry, Synovis, St Paul, MN) through a randomized controlled trial [19]. The CR-POPF rate was significantly lower in the mesh reinforcement group than in the staple alone group (1.9% vs. 20%, *p* = 0.0007). Hassenpflug et al. investigated the benefit of covering the resection margin by a teres ligament patch [20]. In their study, although the overall CR-POPF rate was not reduced by the covering method (32.9% vs. 22.4%, *p* = 0.200), the coverage of the pancreatic remnant after DP was associated with less morbidities. Akca et al. introduced a method of covering the resection margin after DP with the gastric wall or omentum majus [21]. The no-cover group showed a higher frequency of POPF than the cover group did (22% vs. 10%, *p* > 0.05). In a prospective multicenter randomized controlled study, Park et al. utilized TachoSil (a biological sealing patch, Nycomed GmbH, Linz, Austria) to prevent the occurrence of POPF after DP [8]. There was no difference in the overall incidence of CR-POPF between the control group and TachoSil group (28.3% vs. 22.9%, *p* = 0.536).

This study has several limitations. First, this is a retrospective study and not a randomized controlled trial. Given that the method of wrapping the PGA sheet on the pancreas first and then stapling is a newly attempted technique in our clinical practice since November 2018, the changes in the surgical procedures over time should be considered. However, since all cases were performed by one surgeon, the selection of Endo-GIA cartridge according to the pancreatic thickness and texture and the type and location of closed suction drains inserted into the surgical field were applied equally to all patients. Given that these factors were important when assessing the presence of POPF or POFC, we were able to minimize the procedure-related bias. Second, we did not accurately measure the volume of fluids that actually accumulated in the abdominal cavity. For the measurement of fluid volume, the length and width on the axial CT image and the height on the coronal CT image were measured as a hexahedral value; thus, it does not completely represent the actual volume of fluid in a real irregular shape. If volumetry was used, the exact fluid volume could have been measured, but since the same measurement method was applied to all patients, the comparison of fluid volumes between the two groups is appropriate. Third, even though the amount of POFC on POD 7 was lower in the Before-stapling group than in the After-stapling group, there were no statistical differences in the rates of re-intervention or the natural course of spontaneous resolution of asymptomatic POFC between the two groups. Based on these results, this study failed to show advantages of the Before-stapling technique other than the lower amount of POFC. Fourth, this study enrolled a relatively small number of patients. Although 44 patients were enrolled in this study, which was more than the number of patients enrolled in previous studies (22 [17] and 26 [9] patients) using a similar method, it is still a small number of patients. Lastly, the results of this study are difficult to generalize, since all surgical procedures were performed by a single surgeon. Further research is needed in the future to prove the feasibility and effectiveness of Before-stapling method with randomized control trial in large patients group.

In conclusion, applying a PGA sheet to the resection line before or after stapling during DP was an effective method to reduce the incidence of CR-POPF, although no difference was observed between the Before- and After-stapling groups. Even though most cases of fluid accumulation occurring in the surgical field after DP were of no clinical significance, applying a PGA sheet before stapling was a simple and effective way to reduce the risk of fluid collection on the pancreatic stump.

## Supplementary Information

Below is the link to the electronic supplementary material.

Supplementary file1 (DOCX 18 KB)
