# Supplementary material for: Wrapping the pancreas with a polyglycolic acid sheet before stapling reduces the risk of fluid collection on the pancreatic stump after distal pancreatectomy
Source: Surg Endosc. 2021 Feb 23;36(2):1191–8. doi: 10.1007/s00464-021-08387-0 (PMC8758620; doi:10.1007/s00464-021-08387-0)
Supplement: Supplementary file 1 — Supplementary file1 (DOCX 18 KB) [file 464_2021_8387_MOESM1_ESM.docx]

**Supplementary Table.** Analysis of risk factors affecting the clinically relevant POPF

| Factors | Univariate analysis | | | Multivariate analysis | | |
| --- | --- | --- | --- | --- | --- | --- |
|  | *HR* | *95% CI* | *p-value* | HR | 95% CI | *p-value* |
| Age |  |  |  |  |  |  |
| < 58.3 | 1 | - | - | 1 | - | - |
| ≥58.3 | 0.832 | 0.201–3.432 | 0.799 | 0.680 | 0.146–3.166 | 0.623 |
| Sex |  |  |  |  |  |  |
| Male | 1 | - | - | 1 | - | - |
| Female | 0.113 | 0.014–0.942 | 0.044 | 0.138 | 0.016–1.228 | 0.076 |
| BMI |  |  |  |  |  |  |
| < 22.5 | 1 | - | - | 1 | - | - |
| ≥22.5 | 0.374 | 0.073–1.904 | 0.236 | 0.445 | 0.078–2.528 | 0.361 |
| Malignant |  |  |  |  |  |  |
| No | 1 | - | - | 1 | - | - |
| Yes | 1.149 | 0.266–4.962 | 0.853 | 0.793 | 0.144–4.370 | 0.790 |
| Method of surgery |  |  |  |  |  |  |
| Open surgery | 1 | - | - | 1 | - | - |
| Laparoscopic surgery | 0.150 | 0.018–1.242 | 0.078 | 0.127 | 0.014–1.696 | 0.127 |
| Spleen preservation |  |  |  |  |  |  |
| No | 1 | - | - | 1 | - | - |
| Yes | 0.422 | 0.051–3.527 | 0.426 | 0.689 | 0.065–7.262 | 0.756 |
| Location of pancreatic resection |  |  |  |  |  |  |
| Body to tail | 1 | - | - | 1 | - | - |
| Neck | 0.851 | 0.206–3.513 | 0.824 | 0.552 | 0.094–3.257 | 0.512 |
| Operative time |  |  |  |  |  |  |
| < 284.0 | 1 | - | - | 1 | - | - |
| ≥284.0 | 1.096 | 0.266–4.524 | 0.899 | 0.581 | 0.099–3.414 | 0.548 |
| Intraoperative blood loss |  |  |  |  |  |  |
| < 284.5 | 1 | - | - | 1 | - | - |
| ≥284.5 | 2.053 | 0.495–8.506 | 0.321 | 1.236 | 0.220–6.946 | 0.810 |
| Group |  |  |  |  |  |  |
| After-stapling | 1 | - | - | 1 | - | - |
| Before-stapling | 1.048 | 0.204–5.387 | 0.956 | 1.079 | 0.170–6.839 | 0.936 |
